# High Altitude as a Risk Factor for Venous Thromboembolism in Tibial Plateau Fractures

**DOI:** 10.7759/cureus.24388

**Published:** 2022-04-22

**Authors:** Corey A Jones, Matthew S Broggi, Jeffrey S Holmes, Erik B Gerlach, Cody J Goedderz, Shadman H Ibnamasud, Roberto Hernandez-Irizarry, Mara L Schenker

**Affiliations:** 1 Department of Orthopaedic Surgery, Emory University, Atlanta, USA; 2 Department of Orthopaedics, Northwestern University Feinberg School of Medicine, Chicago, USA; 3 Department of Orthopaedic Surgery, Medical College of Georgia, Augusta University, Augusta, USA

**Keywords:** pulmonary embolism, deep venous thrombosis, venous thromboembolism, altitude, tibial plateau fracture

## Abstract

Background*:* Tibial plateau fractures are often significant injuries that can require complex surgical interventions with prolonged perioperative immobilization, thereby increasing the risk of developing venous thromboembolic (VTE) events, specifically, deep vein thrombosis (DVT) and pulmonary embolism (PE). Risk stratification is paramount for guiding VTE prophylaxis. Although high altitude has been suggested to create a prothrombotic state, virtually no studies have explored its clinical effects in lower extremity trauma. The purpose of this study was to compare surgical fixation of tibial plateau fractures at high and low altitudes and its effects on post-operative VTE development.

Methods*:* The Truven MarketScan claims database was used to retrospectively identify patients who underwent surgical fixation of isolated and closed tibial plateau fractures using Current Procedural Terminology (CPT) codes over a 10-year period. Extraneous injuries were excluded using the International Classification of Diseases, 10^th^ edition (ICD-10), and CPT codes. Patient demographics, comorbidities, and DVT chemoprophylaxis prescriptions were obtained. Patients were partitioned into high altitude (>4000 feet) or low altitude (<100 feet) cohorts based on the zip codes of their surgery locations. One-to-one matching and univariate analysis were used to assess and control any baseline discrepancies between cohorts; multivariate regression was then performed between cohorts to determine the odds ratios (OR) for developing VTEs post-operatively.

Results*:* There were 7,832 patients included for analysis. There was no statistical difference between high and low altitude cohorts in developing VTEs within 30 days post-operatively. Higher altitudes were associated with increased odds of developing DVT (OR 1.21, p = 0.043) and PE (OR 1.27, p = 0.037) within 90 days post-operatively.

Conclusions*:* Surgical fixation of tibial plateau fractures is associated with an increased risk of developing VTEs at high altitudes within 90 days post-operatively. Understanding such risk factors in specific orthopaedic patient populations is essential for optimizing DVT prophylaxis protocols. Further studies should investigate this relationship and the role of DVT prophylaxis regimens in this population.

## Introduction

Perioperative venous thromboembolism (VTE), including deep vein thromboembolism (DVT) and pulmonary embolism (PE), continues to be a subject of debate and investigation in the orthopedic literature. A considerable amount of the current evidence on DVT and PE stems from elective arthroplasty or hip and pelvis trauma populations [1-4]. Orthopedic trauma patients are at particular risk with their often sub-optimized medical comorbidities, associated injuries, perioperative weight-bearing restrictions, and systemic inflammatory state. Specific risk factors, optimal chemoprophylaxis, and duration of therapy are less concrete in isolated lower extremity orthopedic trauma patients, and current practices vary substantially across the United States [5].

The reported incidence of DVT and PE in the trauma population has been reported to be as high as 80%, with well-documented morbidity and mortality [6,7]. Unlike hip and pelvis trauma, isolated lower extremity injuries and their propensity for DVT and PE have a relative scarcity of data and vague recommendations for prophylaxis. Tibial plateaus, which account for 1%-2% of adult fractures, confer significant morbidity due in part to their long period of immobilization, mechanism, prolonged hospital admissions, and association with compartment syndrome [8-10]. Smaller-scale studies have reported VTE incidences ranging from .03% to 50% of VTE in tibial plateau fracture populations [11-13]. Virtually no large-scale, longitudinal data exist on the specific risk profiles associated with perioperative DVT or PE in isolated, operative tibial plateau fracture populations.

In order to optimize DVT prophylaxis protocols, a thorough understanding of the risk factors unique to each patient’s fracture morphology must be sought out and available. Multiple studies have been published on risk factors that increase a trauma patient’s risk for VTE events [3,5,14]. However, despite higher altitudes having been shown to lead to adaptive changes that favor a prothrombotic environment, there are few publications evaluating its association in orthopedic trauma populations [2,15,16]. Interestingly, arthroscopy, arthroplasty, and spine fusion literature show higher rates of symptomatic VTE in patients undergoing surgery at higher elevations when compared to lower elevations [17-21]. These authors suggest that physiological adaptations in higher elevation populations may predispose these patients to VTE. The aim of this study was to analyze altitude as a risk factor for VTE in isolated, operative tibial plateau fractures. We hypothesize that higher altitudes would increase the risk of VTE in the tibial plateau population.

## Materials and methods

Data source and selection

This was a retrospective investigation of patients with isolated and closed tibial plateau fractures that received operative intervention in the United States between January 1, 2009 and December 31, 2019. The Truven Health MarketScan database was utilized for patient identification and selection. This database contains healthcare utilization data on more than 240 million patients through private insurance claims, including Medicare. These records allow for the analysis of multiple parameters, including procedure data, location, medical comorbidities, medical prescriptions, and post-operative follow-up care. Prior orthopedic studies have used this database for clinical investigation and it is well-published in the literature [2]. 

We used common Current Procedural Terminology (CPT) codes to query the database for patients who underwent surgical fixation of tibial plateau fractures (Table 1). Our exclusion criteria included: open fracture(s), patients currently prescribed oral contraceptives, patients who were not continuously enrolled in the database for six months prior to or after surgery, prior history of VTE, hypercoagulable medical condition (defined as having an International Classification of Diseases (ICD) code for Factor V Leiden, antiphospholipid antibody syndrome and/or Protein C and S deficiency), and less than 18 years of age at the time of surgery. In line with previous studies, we expanded our query to six months to ensure adequate follow-up and to exclude patients who might have a predisposition to developing a VTE [2]. Additionally, because tibial plateau fractures commonly occur in the polytraumatic patient, we attempted to control for, and exclude, patients with co-existing injuries to reduce potential confounders. The ICD and CPT codes used to isolate tibial plateau fractures are provided in Tables 2-3. 

**Table 1:** Tibial Plateau ORIF CPT Codes ORIF: open reduction internal fixation; CPT: current procedural terminology.

CPT Code	Description
27535	Open treatment of tibial fracture, proximal (plateau); unicondylar, includes internal fixation when performed
27536	Open treatment of tibial fracture, proximal (plateau); bicondylar, with or without internal fixation

**Table 2:** CPT Exclusion Codes CPT: current procedural terminology.

CPT Code	Description
49000	Ex-Lap
49320	Diagnostic Ex-Lap
32551	Chest tube placement
32150	Thoracotomy with exploration
31500	Rapid sequence intubation
37244	REBOA
33025	Pericardial window
75630	Angiography of lower extremity
27245	Inter, per, subtroch femur fracture with IMN
27506	Femoral shaft Fx w/ IMN
27507	Femoral shaft Fx w/ plates and screws
27514	Open treatment of femoral fracture, distal end, medial or lateral condyle, includes internal fixation when performed
27513	Open treatment of femoral supracondylar or transcondylar fracture with intercondylar extension, includes internal fixation when performed
27758	Open treatment of tibial shaft fracture (with or without fibular fracture), with plates/screws, with or without cerclage
27759	Treatment of tibial shaft fracture (with or without fibular fracture) by intramedullary implant, with or without interlocking screws and/or cerclage
27766	Open treatment of medial malleolus fracture, includes internal fixation when performed
27769	Open treatment of posterior malleolus fracture, includes internal fixation when performed
27792	Open treatment of distal fibular fracture (lateral malleolus), includes internal fixation when performed
27814	Open treatment of bimalleolar ankle fracture (e.g., lateral and medial malleoli, or lateral and posterior malleoli, or medial and posterior malleoli), includes internal fixation when performed
27822, 27823	Open treatment of trimalleolar ankle fracture, includes internal fixation when performed, medial and/or lateral malleolus; with or without fixation of posterior lip
27827	Open treatment of fracture of weight bearing articular surface/portion of distal tibia (e.g., pilon or tibial plafond), with internal fixation when performed; of tibia only
27828	Open treatment of fracture of weight bearing articular surface/portion of distal tibia (e.g., pilon or tibial plafond), with internal fixation when performed; of both tibia and fibula
27848	Open treatment of ankle dislocation, with or without percutaneous skeletal fixation; with repair or internal or external fixation
28400	Closed treatment of calcaneal fracture; without manipulation
28415	Open treatment of calcaneal fracture, includes internal fixation when performed
28615	Open treatment of tarsometatarsal joint dislocation, includes internal fixation when performed
28445	Open treatment of talus fracture, includes internal fixation when performed

**Table 3:** ICD-10 Exclusion Codes ICD-10: International Classification of Diseases, Tenth Revision

ICD-10 Code	Description
S06	Intracranial Injury
S09.90	Unspecified injury to the head
S02.0, S02.1	Fracture of the skull
S02.8, S02.91	Fracture of other specified skull and facial bones, unspecified fracture
I31.3	Pericardial effusion
I31.4	Cardiac tamponade
S27.32	Lung contusion
J94.2	Hemothorax
J93.9	Pneumothorax
S22.49XA	Multiple rib fractures, unspecified side
S36.113A	Laceration of liver, unspecified degree, initial encounter
S36.03	Splenic laceration
S37.03	Renal laceration
S14.109A	Spinal cord injury
J12.82	COVID-19 Pneumonia
Z86.16	Personal history of COVID-19

Baseline and geographic patient data 

Patient data was obtained to include baseline characteristics such as biological sex, age, and co-morbid medical conditions; these included obesity, renal disease, hypertension, hyperlipidemia, tobacco use, coronary artery disease, diabetes, and cardiac dysrhythmias. Information regarding the severity of their fracture (unicondylar or bicondylar) was also obtained. In addition, the type of post-operative thrombotic chemoprophylaxis, if prescribed, was included for analysis.

Lastly, the database allowed us to procure the locations of the operative procedures for each respective patient. A commercially available zip code database (Datasheer, L.L.C., Hope Junction, New York) was then used to determine each patient’s altitude at the time of surgery. In line with previous literature, altitude was divided into two cohorts: high-altitude (4,000 feet or greater) or low-altitude (100 feet or less) with the assumption that a patient’s post-operative course occurred at the same altitude as their original operation [2,20]. 

Primary outcomes

The primary outcome was post-operative VTE development (DVT, PE, or the combination of both a DVT and PE) at 30 and 90 days.

Statistical analysis

After patient identification, inclusion, and cohort partitioning, we performed one-to-one matching based on patient age and sex. Subsequent analysis was then performed on matched cohorts. Univariate analysis using student t-test and Chi-squared test, when appropriate, was performed to examine initial discrepancies in baseline characteristics and comorbidities between cohorts. To further determine the effects of altitude while controlling for comorbidities and demographic variables, binomial multivariate logistic regression analysis was performed between cohorts. Results are reported as odds ratios (OR) with 95% confidence intervals (CI) and a p-value of less than .05 was considered significant. All statistical analysis was performed using SAS (SAS 9.4, Cary, North Carolina) statistical software.

## Results

Baseline characteristics and comorbidities 

A total of 7,832 patients who underwent surgical fixation of unicondylar and bicondylar tibial fractures were included in the analysis. There were an equal number of patients in both high altitude and low altitude groups (Table 4). There were no statistical differences between the cohorts for age, sex, post-operative DVT prophylaxis, or fracture type. The patient group undergoing fixation at low altitude had a higher rate of tobacco use (9.8% vs. 7.4%, p = 0.047) compared to the patients at high altitude. The full list of comorbidities and their differences are shown in Table 4.

**Table 4:** Baseline Patient Characteristics and Comorbidities n: number; %: percentage; avg: average; SD: standard deviation; LMWH: low molecular weight heparin; DOAC: direct oral anticoagulant.

	High Altitude	Low Altitude	P-Value
Total, n	3,916	3,916	
Age, avg (SD)			
18-25	365 (9.3)	365 (9.3)	0.999
26-35	474 (12.1)	474 (12.1)	0.999
36-45	661 (16.9)	661 (16.9)	0.999
46-55	997 (25.5)	997 (25.5)	0.999
56-65	909 (23.2)	909 (23.2)	0.999
+65	510 (13.0)	510 (13.0)	0.999
Sex, n (%)			
Male	2,259 (57.7)	2,259 (57.7)	0.999
Female	1,657 (42.3)	1,657 (42.3)	
Tibial Plateau Fracture			
Unicondylar	3,412 (87.1)	3,412 (87.1)	0.345
Bicondylar	504 (12.9)	504 (12.9)	
Post-operative DVT Prophylaxis			
Aspirin	1,467 (37.5)	1,446 (36.9)	0.224
LMWH	1,418 (36.2)	1,479 (37.8)	0.201
DOAC	538 (13.7)	495 (12.6)	0.109
Other	369 (9.4)	340 (8.7)	0.272
Comorbidities, n (%)			
Obesity	838 (21.4)	896 (22.9)	0.532
Renal Disease	44 (1.1)	39 (1.0)	0.471
Hyperlipidemia	1,046 (26.7)	1,013 (25.9)	0.328
Tobacco Use	289 (7.4)	383 (9.8)	0.047
Hypertension	1,283 (32.8)	1,340 (34.2)	0.088
Congestive Heart Failure	67 (1.7)	70 (1.8)	0.495
Coronary Artery Disease	326 (8.3)	340 (8.7)	0.362
Cardiac Dysrhythmia	36 (0.9)	27 (0.7)	0.322
Diabetes	708 (18.1)	774 (19.8)	0.477

Altitude and post-operative VTE

In the 30 day post-operative time period, high-altitude surgery was associated with VTEs. More high-altitude patients developed DVT compared to the low-altitude patients (0.48% vs 0.33%, p = 0.039). Within 90 days post-operatively, more high-altitude patients developed either a DVT (0.70% vs 0.53%, p = 0.037) or PE (0.41% vs 0.27%, p = 0.029) compared to the low-altitude cohort (Table 5). 

**Table 5:** Univariate Analysis of Altitude and DVT and/or PE n: number; %: percentage; DVT/PE: deep vein thrombosis and/or pulmonary embolism; VTE: venous thromboembolism.

VTE, n (%)	High Altitude	Low Altitude	P-Value
30-days postoperatively			
Deep vein thrombosis (DVT)	19 (0.48)	13 (0.33)	0.039
Pulmonary embolism (PE)	8 (0.19)	7 (0.18)	0.724
DVT and PE	7 (0.17)	8 (0.19)	0.651
90-days postoperatively			
Deep vein thrombosis (DVT)	28 (0.70)	21 (0.53)	0.037
Pulmonary embolism (PE)	29 (0.41)	19 (0.27)	0.029
DVT and PE	8 (0.21)	10 (0.24)	0.373

On multivariate analysis, the development of DVT (OR 1.18, CI 0.98-1.31) or PE (OR 1.15, CI 0.88-1.44) at 30 days did not have a significant association with altitude. At 90 days post-operatively, the development of DVT (OR 1.21, CI 1.06-1.47) or PE (OR 1.27, CI 1.11-1.54) had a significant association with altitude (Table 6). At both 30 and 90 days, the development of both a DVT and a PE was not statistically different between cohorts in both univariate and multivariate analysis. 

**Table 6:** Multivariate Analysis of Altitude and DVT/PE* *Model controlled for all demographic variables listed in Table 2. Reference group: low altitude cohort. OR: odds ratio; CI: confidence interval; VTE: venous thromboembolism; DVT/PE: deep vein thrombosis and/or pulmonary embolism.

VTE	OR	95% CI	P-Value
30-days postoperatively			
Deep vein thrombosis (DVT)	1.18	0.98-1.31	0.097
Pulmonary embolism (PE)	1.15	0.88-1.44	0.572
DVT and PE	1.08	0.82-1.38	0.627
90-days postoperatively			
Deep vein thrombosis (DVT)	1.21	1.06-1.47	0.043
Pulmonary embolism (PE)	1.27	1.11-1.54	0.037
DVT and PE	1.07	0.89-1.34	0.316

## Discussion

Our study demonstrated in a large, multicenter population that patients undergoing surgical fixation of tibial plateau fractures at higher altitudes are at an increased risk for DVT and PE peri-operatively. Within 90 days post-operatively, patients in high altitudes were 1.21 times more likely to form a DVT and 1.27 times more likely to form a PE compared to those in low altitudes.

It is well known that high altitudes induce physiological changes for adaption to a relatively lower oxygen tension environment. Previous literature has shown that these changes may predispose to thrombogenesis by way of hypercoagulability and venous stasis [2,15,16]. As the oxygen tension lowers at higher altitudes there is a compensatory increased red blood cell and resulting hemoconcentration [2,17]. This can cause a hypercoagulable state with an increase in clotting factors which later may progress into a hyper-fibrinogenic state and platelet dysfunction for as long as the person is exposed to high altitude [22-24]. A study by Kotwal et al. that looked at a cohort of patients at 3500m showed that the milieu of platelet activation, fibrinogen increase, and dehydration led to thrombotic cases in even normal individuals [25]. These changes induced by high altitude environments, in addition to the trauma and prolonged immobilization often seen in tibial plateau fractures, likely subjects these patients to an increased risk for VTE. 

Orthopaedic patients are at particular risk of DVT and the effects of high altitude have only recently been explored in this population. Damodar et al. showed that patients undergoing elective total hip arthroplasty were 1.74 and 1.60 times more likely to develop a PE at 30 and 90 days post-operatively at high altitudes compared to lower altitudes [17]. A study by Tyson et al. showed a 3.8 times increased risk of VTE in patients undergoing knee arthroscopy at higher altitudes as compared to lower altitudes [19]. Additional studies have demonstrated similar results in patients undergoing total shoulder arthroplasties and lumbar spine fusions [21,26]. Despite these findings in elective surgical settings, there is a scarcity of investigation in the orthopedic trauma literature whose population is at particular risk for VTE. During our literature review, only one study investigated altitude and VTE risk in orthopedic trauma populations. Broggi et al. showed that patients with acetabular and pelvic ring injuries were 1.47 and 1.63 times more likely to develop a DVT and PE, respectively, at 90 days when surgically treated at higher altitudes [2]. Tibial plateau fractures are known to have a lower overall incidence of VTEs as compared to the pelvis and hip fractures, which is consistent with the results of this study [1,3,9,12,14,27]. In addition, it is unclear if concurring injuries were excluded in the formers analysis, which may potentially confound their results. The present study is among the first to publish on altitude as a risk factor for VTE in orthopedic trauma populations and, to our knowledge, is the first to publish on this subject in tibial plateau fracture populations. 

Our results found that at 30 days post-operatively from tibial plateau fixation, patients had a similar risk of VTE in both high and low altitude operative settings. It was not until 90 days post-operatively that higher altitude patients had a significantly elevated risk of VTE. These results are similar to those published by Broggi et al., which found that patients undergoing traumatic pelvis and/or acetabular surgery were at increased risk of VTE at 90 days as compared to 30 days in high altitudes. A rationale for these findings is that the physiologic changes adopted at high altitudes can occur over weeks to months [28]. Therefore, altitude itself may be a subtle risk factor that appears to expand the perioperative time window in which trauma patients are susceptible to VTE. Current guidelines for DVT prophylaxis based on isolated tibial plateaus is scarce, with most recommendations based on literature from the hip and pelvis fracture population [1,3,5,14]. It is no surprise then that the DVT chemoprophylaxis patterns vary widely throughout the world and country [29,30]. This information, in our fracture-specific population, may help to equip practitioners to better risk stratify and educate patients about their individual risk and guide DVT chemoprophylaxis regimen and duration. 

There are several limitations to our study. The database used does not specify how the VTEs were diagnosed (i.e. screening, Doppler ultrasound, venography, clinically, etc.). It is also unknown how these events may have contributed to patient morbidity and mortality. However, there is no reason to believe that these strategies would differ between regions. It also does not give us information on the specific dosages of VTE prophylaxis or the duration of prophylaxis used amongst providers. In an attempt to control for post-operative VTE chemoprophylaxis, we included concomitant prescriptions for the most common chemoprophylaxis (aspirin, direct oral anticoagulation, and low molecular weight heparin) which were similar between groups [5,30]. We attempted to isolate tibial plateau fractures by use of various procedural exclusion codes; however, this list is not exhaustive. We relied on accurate procedural coding of the surgeries and comorbidities which is intrinsically prone to human error. This would have caused us to possibly under-capture the VTE rate, although the error rate in both cohorts should theoretically be similar. While the study controlled for a plethora of baseline patient characteristics, other uncapturable risk factors such as hormonal therapy, family history, and prior VTE could have been confounding but likely similar in both groups. In addition, the database was unable to report on the mechanism of injury, specific fracture morphologies, time until definitive fixation, surgical time, single vs. dual plating, and did not specify if and how many patients may have been initially managed in external fixation prior to definitive fixation, as well as patients definitively managed in external fixation. Despite these limitations, this multicenter, national database allows us to investigate large population data. Given the relatively low incidence of tibial plateau fractures and associated VTE, it would be difficult to thoroughly investigate these otherwise subtle risk factors for VTE that might go missed in smaller, single-center series. 

## Conclusions

The current study found that there was a significantly increased risk of VTE in post-operative tibial plateau patients who were treated at high altitudes. Understanding such risk factors in specific orthopaedic patient populations is essential for optimizing DVT prophylaxis protocols. Further investigation into this relationship and effects of both regiment and duration of chemoprophylaxis should be sought.
